# A single-stage bilayered skin reconstruction using Glyaderm® as an acellular dermal regeneration template results in improved scar quality: an intra-individual randomized controlled trial

**DOI:** 10.1093/burnst/tkad015

**Published:** 2023-05-02

**Authors:** Ignace De Decker, Henk Hoeksema, Jozef Verbelen, Petra De Coninck, Marijn Speeckaert, Sofie De Schepper, Phillip Blondeel, Ali Pirayesh, Stan Monstrey, Karel E Y Claes

**Affiliations:** Burn Center, Ghent University Hospital, C. Heymanslaan 10, 9000 Ghent, Belgium; Department of Plastic Surgery, Ghent University Hospital, C. Heymanslaan 10, 9000 Ghent, Belgium; Burn Center, Ghent University Hospital, C. Heymanslaan 10, 9000 Ghent, Belgium; Department of Plastic Surgery, Ghent University Hospital, C. Heymanslaan 10, 9000 Ghent, Belgium; Burn Center, Ghent University Hospital, C. Heymanslaan 10, 9000 Ghent, Belgium; Burn Center, Ghent University Hospital, C. Heymanslaan 10, 9000 Ghent, Belgium; Department of Nephrology, Ghent University Hospital, C. Heymanslaan 10, 9000 Ghent, Belgium; Department of Dermatology, Ghent University Hospital, C. Heymanslaan 10, 9000 Ghent, Belgium; Burn Center, Ghent University Hospital, C. Heymanslaan 10, 9000 Ghent, Belgium; Department of Plastic Surgery, Ghent University Hospital, C. Heymanslaan 10, 9000 Ghent, Belgium; Plastic surgeon in private practice in Amsterdam, Amsterdam, The Netherlands; Burn Center, Ghent University Hospital, C. Heymanslaan 10, 9000 Ghent, Belgium; Department of Plastic Surgery, Ghent University Hospital, C. Heymanslaan 10, 9000 Ghent, Belgium; Burn Center, Ghent University Hospital, C. Heymanslaan 10, 9000 Ghent, Belgium; Department of Plastic Surgery, Ghent University Hospital, C. Heymanslaan 10, 9000 Ghent, Belgium

**Keywords:** Burn, Scar, Skin, Glyaderm®, Dermal substitute, Dermal regeneration template, Elastin

## Abstract

**Background:**

Absence of almost the entire reticular dermal layer is inherent to the use of autologous split-thickness skin grafting (STSG) to close full-thickness wounds, often resulting in hypertrophic scars and contractures. Many dermal substitutes have been developed, but unfortunately most have varying results in terms of cosmetic and/or functional improvement as well as patient satisfaction, in addition to high costs. Bilayered skin reconstruction using the human-derived glycerolized acellular dermis (Glyaderm®) has been reported to result in significantly improved scar quality using a two-step procedure. Unlike the necessary two-step procedure for most commercially available dermal substitutes, in this study we aimed to investigate the use of Glyaderm® in a more cost-effective single-stage engrafting. This is a method which, if autografts are available, is preferred by the majority of surgeons given the reduction in costs, hospitalization time and infection rate.

**Methods:**

A prospective, randomized, controlled, intra-individual, single-blinded study was performed, investigating the simultaneous application of Glyaderm® and STSG *vs.* STSG alone in full-thickness burns or comparable deep skin defects. During the acute phase, bacterial load, graft take and time to wound closure were assessed and were the primary outcomes. Aesthetic and functional results (secondary outcomes) were evaluated at 3, 6, 9 and 12 months follow-up using subjective and objective scar measurement tools. Biopsies for histological analysis were taken at 3 and 12 months.

**Results:**

A total of 66 patients representing 82 wound comparisons were included. Graft take (>95%), pain management and healing time were comparable in both groups. At 1 year follow-up, the overall Patient and Observer Scar Assessment Scale assessed by the patient was significantly in favour of sites where Glyaderm® was used. Not infrequently, patients attributed this difference to improved skin sensation. Histological analysis showed the presence of a well-formed neodermis, with donor elastin present for up to 12 months.

**Conclusions:**

A single-stage bilayered reconstruction with Glyaderm® and STSG results in optimal graft take without loss of Glyaderm® nor the overlaying autografts due to infection. The presence of elastin in the neodermis was demonstrated during long-term follow-up in all but one patient, which is a crucial factor contributing to the significantly improved overall scar quality as evaluated by the blinded patients.

**Trial registration:**

The trial was registered on clinicaltrials.gov and received the following registration code: NCT01033604.

Highlights

- Simultaneous bilayered skin reconstruction with Glyaderm® results in improved long-term scar quality.
- Single engraftment with Glyaderm® does not reduce the take rate of the overlying autograft.
- Compared to other commercially available substitutes, dermal replacement with Glyaderm® is at low risk for infection and prolonged wound healing.
- Donor elastin fibres could be demonstrated even after 1 year follow-up in all but one patient.
- Compared to other acellular dermal substitutes, Glyaderm® is currently the least expensive option.

## Background

The established treatment of deep partial and full-thickness burns consists of early removal of non-viable tissue followed by skin grafting [1–3]. This approach resulted in mortality reduction in major burns and is essential in modulating the body’s physiologic response, reducing the risk of bacterial colonization and infection and shortening the length of hospital stay [1,4,5]. Inherent to the use of split-thickness skin grafts (STSG) to close these deep defects is the almost complete absence of the deeper dermal layer which often leads to hypertrophic scar (HTS) formation with reported incidences ranging from 32 to 72% post-burn [6–17]. The restoration of normal skin function and cosmesis is the holy grail for every burn surgeon and an important step in achieving this goal is the use of dermal substitutes [18]. Dermal substitutes or dermal regeneration templates (DRTs) aim to improve dermal restoration by providing a neodermis that anatomically functions more like natural dermis rather than fibrotic tissue, therefore improving scar characteristics and improving the patients’ quality of life [5,18]. A wide variety of synthetic and biological dermal substitutes are currently available and they are classified according to scaffold type, thickness, number of layers, cell types, period of application and the type of wound to be treated [19]. A DRT plays the simultaneous role of a supporting structure and an extracellular matrix by providing a scaffold for the formation of a permanently integrated neodermis [4,5,19]. Ideally, dermal templates allow effective fibroblast migration, adequate endothelial cellular influx for the creation of a vascular network, cell proliferation, secretion of native collagen, and the timely degradation and proper formation of new tissue architecture [4,5,19]. The neodermis that creates the framework of the wound needs to be flexible, elastic, able to withstand shear forces and must ensure wound stability for a considerable amount of time [19]. From a surgeon’s perspective, a DRT provides immediate wound coverage post-excision, establishes a barrier preventing fluid loss and allows the use of an ultra-thin autograft, reducing donor site morbidity [5].

Many of the commercially available DRTs focus on supplying a 3D fibre network primarily based on collagen of either xenogenic, allogenic or synthetic origin [20,21]. At the same time, these DRTs are restricted by lack of elasticity and impaired by scaffold contraction [20]. Surprisingly, elastin historically has been inadequately represented in commercial dermal substitutes even though it plays an indispensable role in skin structure and function, mainly determining its resilience, texture and quality [20]. Elastin has inherent cell signalling properties, promoting responses including chemotaxis, cell attachment, proliferation and differentiation and has the potential to limit cellular contractile forces [20,22,23]. Although dermal fibroblasts are inherently capable of secreting the protein monomer elastin, its synthesis is repressed by post-transcriptional mechanisms [24,25]. Moreover, the dermal elastin network does not regenerate adequately after severe wound healing, and even in scars older than a decade, newly synthesized elastin fibres remain fragmented and never reach mature size, correlating with the hard and inelastic nature of HTS [20,26]. Increasing cicatrix quality and especially improving scar elasticity through dermal replacement in the reconstruction of full-thickness skin defects should therefore incorporate a well-preserved 3D collagen–elastin fibre network [27]. A number of collagen–elastin DRTs of human or allogeneic origin are commercially available, e.g. Alloderm®, Dermamatrix®, Surederm® and Glycerolised Acellular Dermis (Glyaderm®, Euro Skin Bank, Beverwijk, The Netherlands) [28,29]. Glyaderm® is preserved in a glycerol solution that has been shown not to harm the skin’s structures and has virucidal properties when incubated and viral particle survival rates that are extremely low [26,30–32]. Irradiation is a different technique of sterilization that only has a minor impact on the antigenicity of the skin and moreover it stiffens and damages the skin by inducing collagen cross-links, impeding the skin from properly adhering to the wound bed due to the creation of free radicals [30,33]. For the storage of tissue, there is also the option of freezing the skin with liquid-phase nitrogen, called cryopreservation. However processing skin with glycerol is simpler, more cost-effective and additionally has antimicrobial and antiviral properties [32].

**Figure 1:**
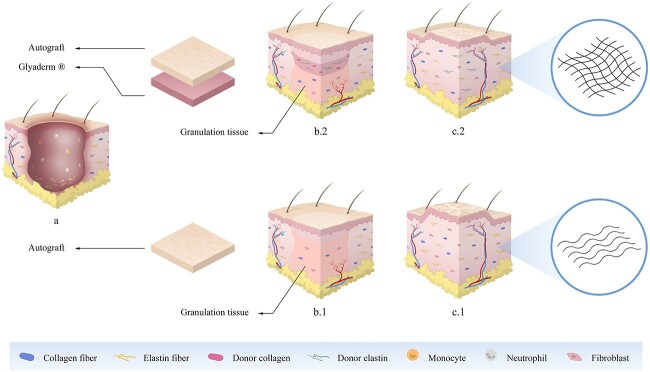
Artistic illustration of the immediate simultaneous bilayered skin reconstruction using Glyaderm® as a dermal substitute on a full-thickness skin defect. (**a**) Defect with epidermal and dermal component involved. (**b.1**) Single-layer reconstruction by autografting without placement of a dermal substitute. (**b.2**) Simultaneous bilayered reconstruction using Glyaderm® and autografts. Subsequent vascularization of the dermal substitute. (**c.1**) Spatial orientation of the fibres is crude and parallel. Scar shows more contracture and hypertrophy compared to the bilayered skin reconstruction. (**c.2**) Spatial orientation of the fibres is similar to the natural basket-weave pattern due to Glyaderm® acting as a guide for infiltrating cells. As a result, the scar shows less contracture and hypertrophy compared to autograft alone

Due to the low-cost incubation and preservation methods, Glyaderm® offers a cost-effective method for dermal substitution in deep partial and full-thickness skin defects. Improvement of scar quality using Glyaderm® as a DRT in a two-step procedure has been demonstrated in a phase III clinical trial including 55 patients [26]. In the study described here, we investigated the use of Glyaderm® in a single-stage setting for the bilayered skin reconstruction of deep or full-thickness burns and comparable skin defects (Figure 1).

## Methods

### Ethics committee

This study was approved by the local ethics committee (B670201733327) and eligible patients were included after obtaining informed consent. Glyaderm® was produced and supplied by the Euro Skin Bank (Beverwijk, The Netherlands). The production process of Glyaderm® has been published by Pirayesh *et al*. [27].

### Study design

This study was a randomized, controlled, single-blind, intra-individual comparison of deep dermal and full-thickness skin defects engrafted simultaneously with Glyaderm® and STSG (intervention) *vs* STSG alone (conventional treatment) in a monocentric setting.

The primary study outcome measures were the evaluation of autologous graft take on days 5–7 post-operative comparing Glyaderm® and STSG* vs* STSG alone, the comparison of healing time between the two procedures and the assessment of the bacterial load. Secondary outcome measures were the functional and aesthetic outcome of a single-stage bilayered skin reconstruction using Glyaderm® and STSG *vs* STSG alone. Secondary outcome measures were evaluated with objective and subjective tools at 3, 6, 9 and 12 months follow-up after achieving wound closure.

### Sample size and patient recruitment

To assess the superiority of single-stage bilayered skin reconstruction with Glyaderm® compared to the reference standard of autologous grafting alone [standard of care (SoC)], assuming a minimal relevant clinical change of 0.05 or 5% improvement in elasticity as measured by the Cutometer Dual MPA 580 (Courage + Khazaka electronic GmbH, Cologne, Germany) and assessed with value *R*2 with an assumed standard deviation of 0.11 and a correlation of 0.39, and based on a paired design and the normally distributed data collected from the previous study, a sample of 75 wound parings being treated with both immediate bilayered skin reconstruction using Glyaderm® and STSG and the reference standard is necessary to achieve 80% power at the 5% significance level.

Patients for this clinical trial were included from the period between February 2017 and August 2020. The last follow-up took place in September 2021. A detailed overview of the eligibility criteria can be found in Table 1.

**Table 1:** Eligibility criteria

**Inclusion criteria**
All deep partial thickness and full-thickness burns as shown by laser Doppler imaging (LDI) and/or clinically evaluated by two plastic surgeons or a burn care coordinator Other full-thickness skin defects besides burns, e.g. necrotizing fasciitis, deglovements or phalloplasty donor sites after free flap harvest Possibility to follow the complete treatment schedule until final graft take and subsequently wound healing and participation in the follow-up schedule Informed consent has been obtained Age between 18–80 years
**Exclusion criteria**
All partial-thickness burns that can heal by conservative treatment confirmed by LDI Not completing the treatment schedule or declining further follow-up The patient has any condition(s) that seriously compromises the patient’s ability to complete this study. The patient has participated in another study utilizing an investigational drug within the previous 30 days The patient has one or more medical condition(s) that, in the opinion of the investigator, would make the patient an inappropriate candidate for this study e.g. diabetes, renal or hepatic insufficiency, immune or neurologic disease

### Surgical regimen

**Figure 2:**
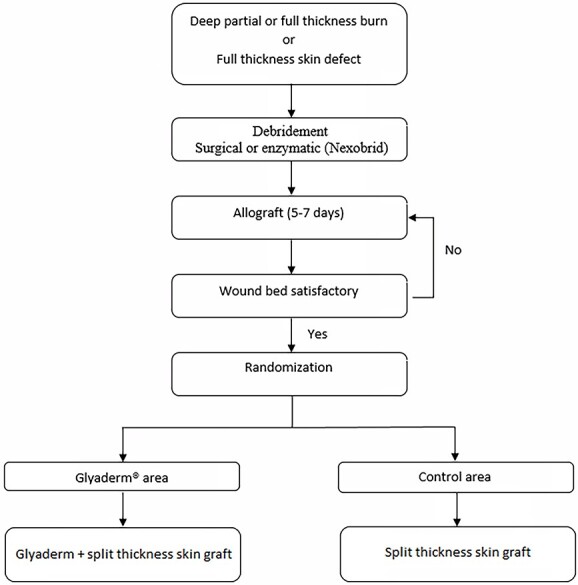
Study flowchart

The regimen of the study is illustrated in Figure 2. Prior to patient enrolment, evaluation of the full-thickness burn wounds or of the other full-thickness skin defects was carried out. Preceding the first operation, the full-thickness wounds were treated according to the burn centre’s local protocol. Burn depth was initially assessed by means of clinical assessment and later (48 h–5 days post-burn) confirmed by laser Doppler imaging (LDI) (Moor-LDI-B2, Moor Instruments Ltd, Axminster, Devon, UK), or with clinical assessment only in the case of clear full-thickness burns and assessed by two plastic surgeons and/or a burn care coordinator. Other full-thickness skin defects in need of skin grafting were eligible, e.g. necrotizing fasciitis, donor site after free radial forearm flap harvest and traumatic deep soft tissue injuries (deglovement injuries).

**Figure 3:**
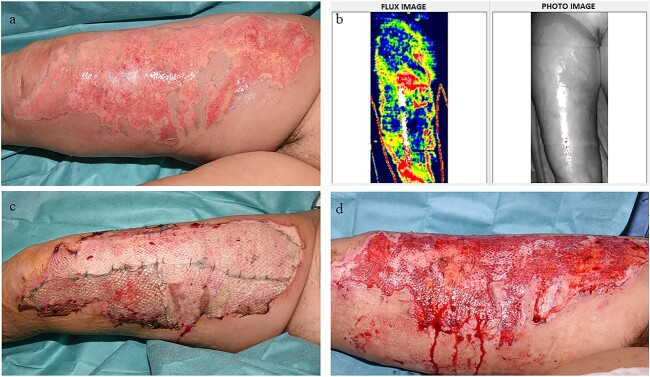
Example case 1. (**a**) A patient admitted with a scald burn (frying oil). (**b**) Burn depth assessment by means of laser Doppler imaging (LDI) on the third day post-burn. LDI blue colour indicates a full-thickness burn. (**c**) Four days after allograft application. (**d**) After removal of allografts and prior to application of Glyaderm® and/or autografting

The first operation consisted of debridement in combination with the application of glycerol-preserved allografts (GPAs; Euro Skin Bank, Beverwijk, The Netherlands) for wound bed preparation (Figure 3). During the second surgical intervention, GPAs were removed and the wound bed was assessed for grafting. If the wound bed was not satisfactory, new GPAs were applied. When deemed suitable for grafting, two comparable wounds or one wound consisting of two comparable parts were randomized into one of the treatment regimens (Figure 2).

### Wound site selection and randomization

The two comparable wound sites were labelled either A or B prior to randomization. In the case of burn injuries, based on LDI, two target wounds (A and B) with comparable healing potential were chosen. In the case of other full-thickness skin defects, such as phalloplasty donor sites where flap harvest is done up to the fascia, two comparable wounds or one wound that could be divided into two comparable wound areas were labelled as target wounds A and B prior to randomization and autografting. Randomization was then performed prior to autografting in the operating room by use of sealed envelopes indicating the treatment regimen per wound site which were made before start of the study. Only moments prior to autografting, the sealed envelope was unsealed to reveal the treatment for each site.

### Procedure A: Glyaderm® + STSG

**Figure 4:**
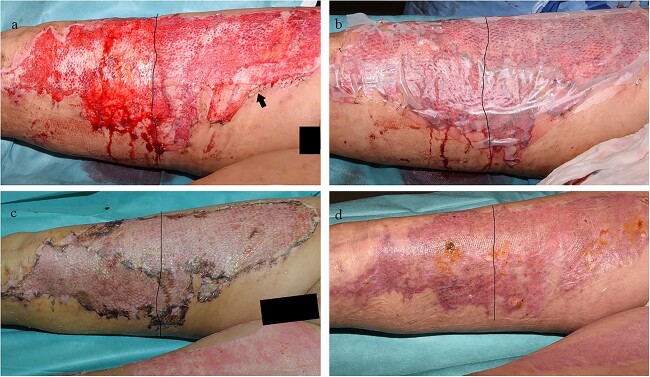
Example case 1. (**a**) Application and fixation of Glyaderm® on the most proximal half of the upper leg. Control and intervention sites are situated left and right of the black line respectively. Arrow indicates Glyaderm® which can be seen as a thin glistening layer. (**b**) Autograft application and coverage with Surfasoft®. (**c**) Removal of Surfasoft® on the sixth day post-autografting. (**d**) Complete wound closure 3 weeks post-autografting

The 85% glycerol-preserved Glyaderm® was rinsed in sterile water for at least 15 min prior to perforation with a special 1 : 1 ratio carrier (Humeca, Borne, The Netherlands). The Glyaderm® was applied and secured with sutures (Figure 4). Subsequently, the Glyaderm® was covered with an autologous STSG (0.012 inches (0.30 mm) thickness/mesh ratio 1 : 1.5; 1 : 2 or 1 : 3) and secured with staples (Figure 4). In the case of phalloplasty donor sites, the autografts were unmeshed and simply perforated using a scalpel. The autograft was then fixed using sutures or staples. The autograft was protected with a semi-permeable membrane: Surfasoft® (Haromed, Ghent, Belgium). The Surfasoft® was covered with a paraffin gauze, povidone-iodine gel and a sterile gauze.

### Procedure B: autograft only

The other wound site was treated with STSG only. The same expansion ratio, fixation methods and wound dressings were used to ensure comparability between both procedures.

### Evaluation during wound healing

Graft take was assessed 7 days post-autografting and scored as a percentage of the total surface area. The pain was assessed at different time intervals ranging from 2 days to 7 weeks post-autografting. Wound swabs for microbial analysis were performed once weekly. Wound swabs are scored on a semi-quantitive scale (−, no growth; + −, scanty; +, light; ++, moderate; +++, heavy) according to the overall bacterial load and the results are subsequently converted to a numerical scale ‘0, 1, 2, 3 and 4’ respectively. Time until complete wound closure, defined as at least 95% epithelialization, was registered.

### Scar treatment after wound closure

Patients all followed our full treatment schedule consisting of early application of pressure garments (at the latest 7–10 days after wound closure), silicones (sheets and garments) and hydration with moisturizers (Alhydran or Dermacress) [14].

### Follow-up regimen

**Figure 5:**
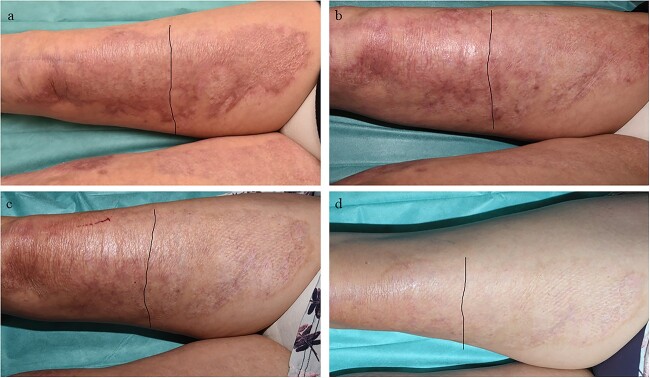
Example case 1. (**a**) Three months after wound closure. (**b**) Six months after wound closure. (**c**) Nine months after wound closure. (**d**) Twelve months after wound closure, the site that received Glyaderm® is more supple, has less contracture and the colour is more normalized compared to the control. Control and intervention sites are situated left and right of the black line respectively.

The patients were seen at the outpatient clinic for evaluation at 3, 6, 9 and 12 months after wound closure (Figure 5). Measurements were taken at all four follow-ups. Elasticity was assessed using the Cutometer dual MPA 580 (Courage + Khazaka electronic GmbH, Köln, Germany). Three parameters were registered: *R*0, *R*2 and *R*8. The *R*0 value assesses the skin’s firmness [34]. The *R*8 parameter represents the ability of the skin to return to its original state after a deformation [34]. The *R*2 parameter can be defined as the ratio of these values ($R2=\frac{R8}{R0}$) and is a parameter for elasticity overall [34]. The average of the elasticity measurements of three random sites of each scar area A and B as well as those of normal skin were used. Also, every individual measurement of these three measurements per site consists of three consecutive measurements, resulting in one average value. Pigmentation and colour were assessed using the Mexameter MX 18 (Courage + Khazaka electronic GmbH, Köln, Germany) with respective parameters erythema index (EI) and melanin index (MI). An average of six measurements all at different sites with the Mexameter was used. Transepidermal water loss (TEWL) was assessed by using a Tewameter TM 300 (Courage + Khazaka electronic GmbH, Köln, Germany). The average of six TEWL measurements of two random sites of the scar site as well as those of normal skin was used. Scar hydration was assessed using a Corneometer CM 825 (Courage + Khazaka electronic GmbH, Köln, Germany). An average of six measurements with the Corneometer, all at different sites, was used. The temperature and humidity of the examination room were always assessed using an ambient condition sensor RHT 100 (Courage + Khazaka electronic GmbH, Köln, Germany).

Both the Patient and Observer Scar Assessment Scale (POSAS) version 2.0 (Dutch Burns Foundation, Beverwijk, The Netherlands) [35] and the Adapted Vancouver Scar Scale (AVSS) were used to subjectively assess scar quality (Supplementary S1 and S2, see online supplementary material) at every follow-up. Patients were blinded throughout the study period because they did not know which area was treated with Glyaderm® and STSG and which with STSG alone.

### Biopsies

Punch biopsies were taken at 3 and 12 months follow-up. Histological analysis was performed by two expert blinded dermatologists (SDS, VV). Automatic hematoxylin and eosin (HE) staining of the paraffin slices was used (T181 Tissue-Tek Prisma Plus, Sakura Finetek, Antwerp, Belgium). To evaluate the collagen and elastin fibre network, Elastica von Giesson staining was used (Benchmark special stains, Roche Diagnostics, Diegem, Belgium). The histological slices were stained using alpha-smooth muscle actin mouse monoclonal antibodies clone BS66 (Nordic Biosite, Täby, Sweden) to evaluate the number of myofibroblasts (Benchmark Ultra ICH/HIS, Roche Diagnostics, Diegem, Belgium). Biopsies were evaluated in terms of collagen and elastin organization, elastin content and dermal aspect, inflammation including the type of white blood cells, organization of blood vessels and number of myofibroblasts. A semi-quantitative scoring system with values ranging from 0–5 was used (Supplementary S3, see online supplementary material). A score of 0 was given to biopsies that resembled normal skin in extracellular matrix structure and cellular presence. A score of 5 was attributed to scar tissue with absence of elastin fibres, strong broadened and eosinophilic collagen strings, pronounced dermal inflammation and overall presence of alpha-smooth muscle actin. Scores of 1–4 represent intermediate values.

### Statistical analysis

Statistical analysis was performed using Graphpad Prism version 9.0.2 (San Diego, CA, USA). The normality of the data was assessed using the Shapiro–Wilk test. Data are presented as mean ± standard deviation (SD). Pairwise comparisons between two groups with normally and non-normally distributed data were assessed with the paired t-test and Wilcoxon matched-pairs signed-rank test, respectively. Pairwise comparisons between more than two groups with normally and non-normally distributed data were detected using the repeated measures one-way analysis of variance (ANOVA) test and Friedman test, respectively. A Geisser–Greenhouse correction was applied for the repeated measures one-way ANOVA due to no assumption of data sphericity. Significant differences between groups were followed by a *post hoc* test. Tukey’s and Dunn’s multiple comparison tests were used for normally (ANOVA) and non-normally (Friedman) distributed data, respectively. *P*-values of <0.05 and < 0.01 were considered *a priori* to be statistically significant and strongly significant, respectively.

## Results

### Patients

**Figure 6:**
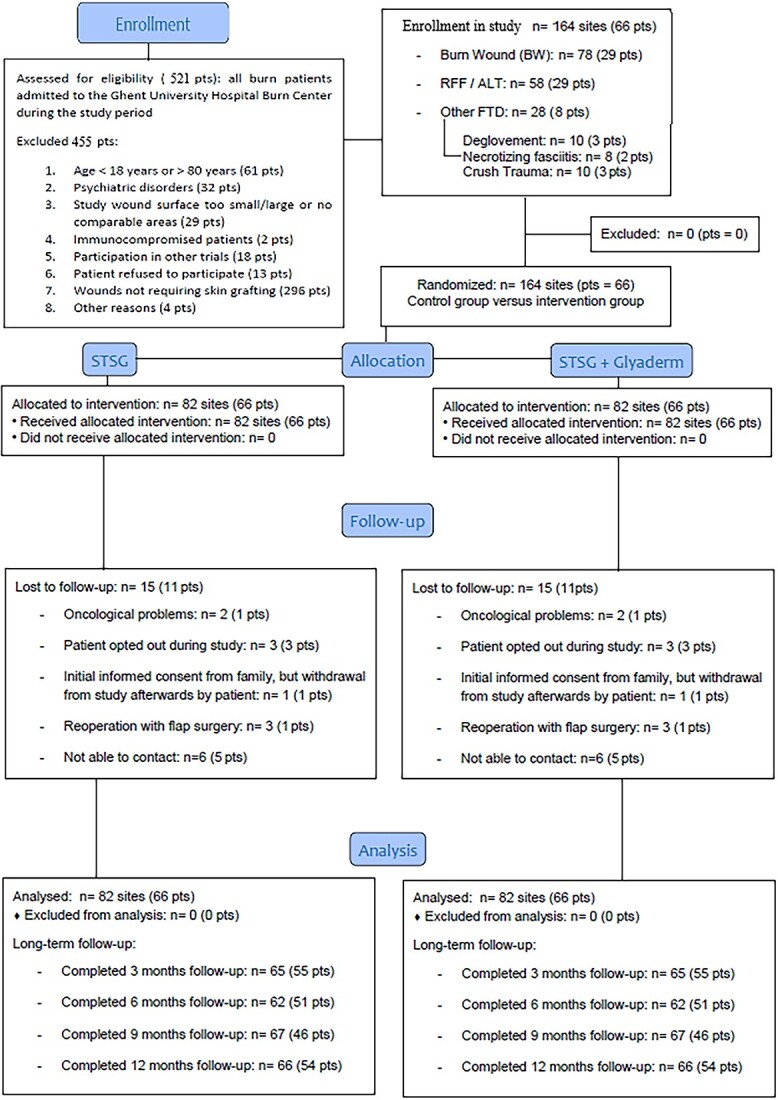
Consort study flowchart of intra-individual study design. *Pts* patients, *RFF* radial forearm flap donor site, *ALT* anterolateral thigh flap donor site, *FTD* full-thickness skin defects, *STSG* split-thickness skin graft

This clinical trial commenced on the 22 February 2017 and ended on the 28 September 2021. A total of 66 patients were included in this intra-individual study, corresponding to 82 wound comparisons. Characteristics of the study population can be found in Table 2 and an overview of the patient recruitment is represented by a Consort flowchart in Figure 6.

**Table 2:** Demographics and patient characteristics

**Grouping**	**Characteristics**	***n* or mean (± SD)**
Patients	Sex, M/F	44/22
	Age	39.47 (±17.99)
	Length	17.94 (±9.65)
	Weight	76.89 (±14.68)
	BMI	25.95 (±4.31)
	Total TBSA%	12.33 (±7.51)
	Burn injuries	29
	Phalloplasty donor site ALT/RFF	29
	Other full-thickness skin defects	8
	Total number of patients	66
Wound aetiology	Burn injuries	39
	Phalloplasty donor site ALT/RFF	29
	Other full-thickness skin defects	14
	Total number of wound comparisons	82
Debridement	Surgical/EDNX	68/14
Graft expansion	Unmeshed	30
	Meshed 1 : 1.5	26
	Meshed 1 : 2	20
	Meshed 1 : 3	5
	Meek 1 : 3	1
Target wounds	TBSA target wound control group	2.30 (±1.87)
	TBSA target wound intervention group	2.35 (±1.94)
	Mean autografts used control group (cm^2^)	144.47 (±112.68)
	Mean autografts used intervention group (cm^2^)	153.53 (±118.56)
	Mean Glyaderm® used intervention group (cm^2^)	177.01 (±127.05)
Wound location control group	Foot left	1
	Gluteal right	1
	Lower arm left	18
	Lower arm right	7
	Lower leg left	8
	Lower leg right	7
	Trunk back	3
	Trunk front	6
	Trunk left	1
	Upper arm left	6
	Upper arm right	5
	Upper leg left	11
	Upper leg right	8
	Total	82
Wound location intervention group	Foot right	1
	Gluteal left	1
	Gluteal right	1
	Lower arm left	18
	Lower arm right	7
	Lower leg left	7
	Lower leg right	10
	Trunk back	3
	Trunk front	4
	Trunk right	1
	Upper arm left	8
	Upper arm right	4
	Upper leg left	9
	Upper leg right	8
	Total	82

*EDNX* Enzymatic debridement with Nexobrid, *RFF* radial forearm flap, *ALT* anterolateral thigh flap, *TBSA* total body surface area, *BMI* body mass index

**Figure 7:**
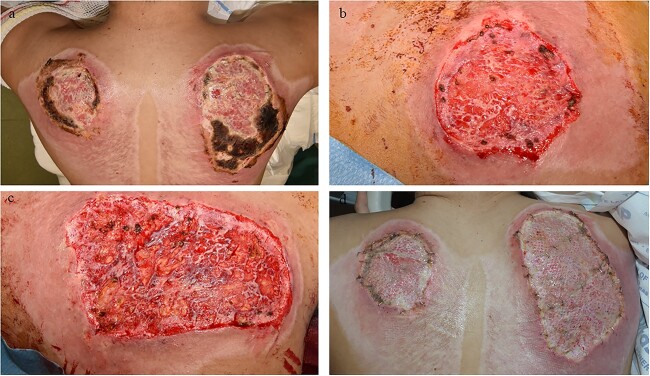
Example case 2. (**a**) Patient admitted with a full-thickness skin defect after a car accident (friction trauma); prior to debridement. (**b**) Left shoulder post-debridement (target wound 1); subsequently, allografts were applied. (**c**) Right shoulder post-debridement (target wound 2); subsequently, allografts were applied. (**d**) Four days after allograft application

**Figure 8:**
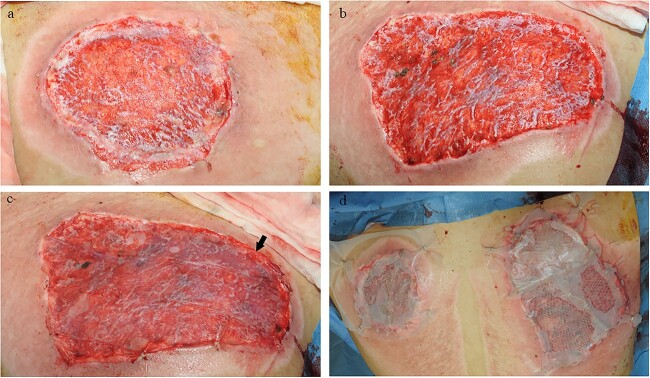
Example case 2. (**a**) Control site (left shoulder) after allograft removal and prior to autografting. (**b**) Intervention site (right shoulder) after allograft removal and prior to Glyaderm® application and autografting. (**c**) Application of Glyaderm® on the right shoulder; arrow indicates Glyaderm®. (**d**) Autografting of both sites(left shoulder control site / right shoulder intervention site) and coverage with Surfasoft®

**Figure 9:**
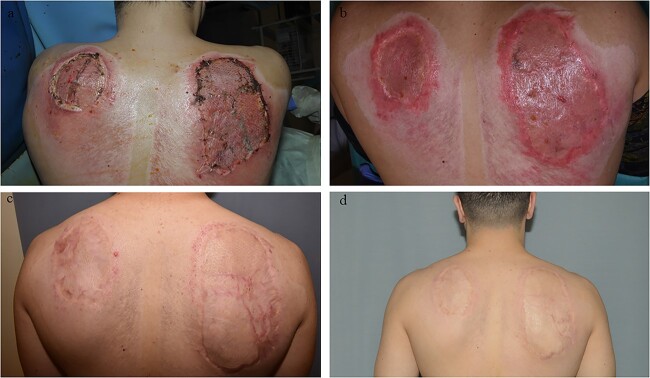
Example case 2. (**a**) Removal of Surfasoft® 6 days post-autografting: left shoulder control; right shoulder intervention. (**b**) Complete wound closure, 3 weeks post-autografting, (**c**) 6 months after wound closure and (**d**) 12 months after wound closure; the site that received Glyaderm® is more supple, has a smoother surface, less hypopigmentation and a more normalized sensation according to the patient

**Figure 10:**
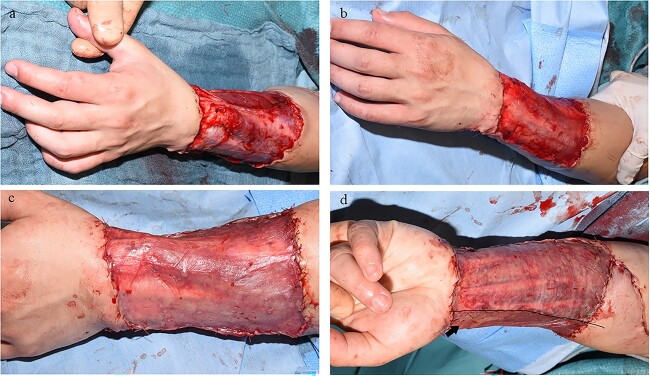
Example case 3. (**a**) Left upper arm after radial forearm flap harvest. (**b**) Dorsal side of the upper arm received Glyaderm®. (**c**) Dorsal side after autograft application onto Glyaderm®. (**d**) Ventral side of left upper arm is covered with autografts only. Dorsal and ventral sides are separated with a black line. A black arrow indicates the side that received Glyaderm®. On the upper side of the black line, the control site is situated (venral side of the left upper arm). On the lower side of the black line, the intervention site is situated (dorsal side of the left upper arm)

**Figure 11:**
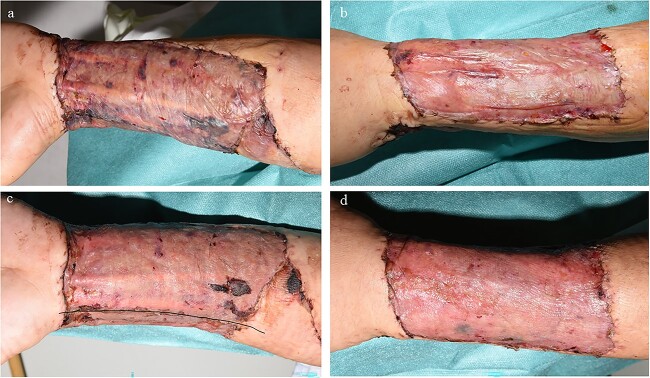
Example case 3. (**a**) Control site 1 week after autografting. (**b**) Glyaderm®-treated site 1 week after autografting. (**c**) Control site 2 weeks after autografting. Black line indicates transition zone. (**d**) Glyaderm®-treated site 2 weeks after autografting. Dorsal and ventral sides are separated with a black line. On the upper side of the black line, the control site is situated (ventral side of the left upper arm). On the lower side of the black line, the intervention site is situated (dorsal side of the left upper arm)

**Figure 12:**
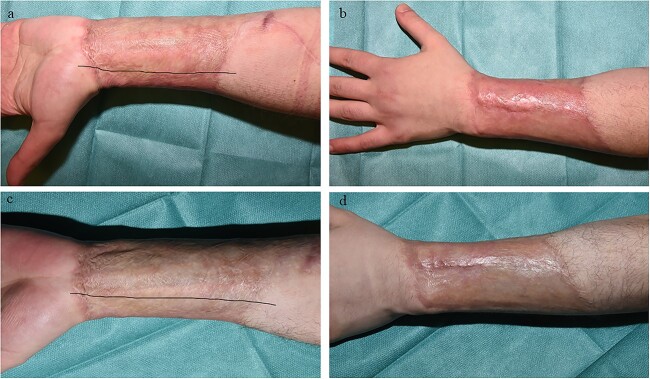
Example case 3. (**a**) Control site 6 months after wound closure. (**b**) Glyaderm®-treated site 6 months after wound closure. (**c**) Control site 12 months after wound closure. Black line indicates transition zone. (**d**) Glyaderm®-treated site 12 months after wound closure. Glyaderm®-treated sites shows less contracture, a smoother appearance and a more favourable colour distribution. Dorsal and ventral sides are separated with a black line. On the upper side of the black line, the control site is situated (ventral side of the left upper arm). On the lower side of the black line, the intervention site is situated (dorsal side of the left upper arm)

The preponderance of male patients is explained by the relatively high number of phalloplasty donor sites included in the study. These patients were considered as male study participants prior to their gender-affirming surgery. Figures 7–9 and 10–12 illustrate two additional example cases of a full-thickness burn and a radial forearm flap donor site, respectively.

### Evaluation in the acute phase

#### Pain

The pain was comparable between both groups (*p* > 0.05) at every moment of evaluation, with the exception of the paint score at 5 weeks which was in fabour of the control site (*p* = 0.031). Mean pain scores, SDs and statistical tests can be found in Supplementary S4, see online supplementary material.

#### Graft take, bacterial load and time to wound closure

Skin graft expansion rates are listed in Table 2. Mean graft take was excellent and comparable in both treatment groups. The graft take was more consistent in the intervention group. Mean graft take was 95.40% (± 10.54%) and 96.22% (± 5.40%) for the control and intervention groups, respectively. No major loss of substitutes or overlying grafts due to inadequate vascularization or infection was seen. Concomitantly, no differences in bacterial load in the weeks post-autografting could be demonstrated on any occasion between both wounds of the wound comparison (*p* > 0.05) based on the regularly obtained wound swabs. At 1 week post-autografting, the mean (±SD) bacterial load of the control and intervention groups were 1.63 (± 0.89) and 1.68 (± 0.92), respectively (*p* = 0.724). At 2 weeks post-autografting this was 1.49 (± 0.91) and 1.55 (± 0.97), respectively (*p* = 0.683). Mean time until complete wound closure was 1.58 (± 0.95) months and was comparable in both groups.

### Long-term evaluation of scar quality

#### Objective measurements

The number of patients, mean values, corresponding SDs, pairwise statistical tests used and complementary statistics of all the objective measurements can be found in Table 3. The multiple comparisons testing can be found in Supplementary S5, see online supplementary material.

##### Corneometer CM 825

Mean (±SD) hydration values of SoC, Glyaderm® and normal skin at 12 months follow-up were 30.40 (±12.43), 30.91 (±13.75) and 30.63 (±12.72) A.U., respectively. There were no differences between groups (SoC, Glyaderm® and normal skin) at 3, 6, 9 and 12 months follow-up.

**Table 3:** Pairwise comparisons, objective measurements and the corresponding data

**Objective measurements**		**Time (months)**	**Group 1 (G1)**	**Group 2 (G2)**	***n***	**Mean (±SD) G1**	**Mean (±SD) G2**	***P*** **-value**	**IQR G1**	**IQR G2**
Mexameter	Erythema	3	STSG	Glyaderm®	54	419.14 (±70.22)	418.62 (±79.64)	0.998^a^	104.74	112.25
			STSG	Normal	54	419.14 (±70.22)	294.03 (±76.09)	<0.0001^*,a^	104.74	124.83
			Glyaderm®	Normal	54	418.62 (±79.64)	294.03 (±76.09)	<0.0001^*,a^	112.25	124.83
		6	STSG	Glyaderm®	57	395.72 (±94.07)	400.31 (±93.49)	>0.9999	150.67	118.34
			STSG	Normal	57	395.72 (±94.07)	291.70 (±85.89)	<0.0001^*^	150.67	93.34
			Glyaderm®	Normal	57	400.31 (±93.49)	291.70 (±85.89)	<0.0001^*^	118.34	93.34
		9	STSG	Glyaderm®	52	373.71 (±94.40)	374.60 (±106.07)	>0.9999	125.94	138.07
			STSG	Normal	52	373.71 (±94.40)	287.92 (±76.65)	<0.0001^*^	125.94	91.83
			Glyaderm®	Normal	52	374.60 (±106.07)	287.92 (±76.65)	<0.0001^*^	138.07	91.83
		12	STSG	Glyaderm®	61	359.14 (±98.64)	368.68 (±86.70)	0.523	102.83	134.17
			STSG	Normal	61	359.14 (±98.64)	291.38 (±84.20)	<0.0001^*^	102.83	110.6
			Glyaderm®	Normal	61	368.68 (±86.70)	291.38 (±84.20)	<0.0001^*^	134.17	110.6
	Pigmentation	3	STSG	Glyaderm®	54	148.74 (±117.38)	223.33 (±113.69)	<0.0001^−^	95.38	86.26
			STSG	Normal	54	148.74 (±117.38)	330.50 (±488.15)	0.0002^*^	95.38	193.25
			Glyaderm®	Normal	54	223.33 (±113.69)	330.50 (±488.15)	>0.9999	86.26	193.25
		6	STSG	Glyaderm®	57	196.70 (±131.45)	187.05 (±123.07)	>0.9999	77.5	96,00
			STSG	Normal	57	196.70 (±131.45)	221.65 (±97.03)	0.002^*^	77.5	78.83
			Glyaderm®	Normal	57	187.05 (±123.07)	221.65 (±97.03)	<0.0001^*^	96,00	78.83
		9	STSG	Glyaderm®	52	199.06 (±140.71)	227.85 (±252.88)	0.842	88.83	95.79
			STSG	Normal	52	199.06 (±140.71)	232.97 (±98.64)	0.032^*^	88.83	93.88
			Glyaderm®	Normal	52	227.85 (±252.88)	232.97 (±98.64)	0.0009^*^	95.79	93.88
		12	STSG	Glyaderm®	61	221.75 (±149.69)	215.68 (±127.37)	>0.9999	117.5	89.67
			STSG	Normal	61	221.75 (±149.69)	238.50 (±115.18)	0.0557	117.5	90.84
			Glyaderm®	Normal	61	215.68 (±127.37)	238.50 (±115.18)	0.033^*^	89.67	90.84
Cutometer	*R*0	3	STSG	Glyaderm®	54	0.61 (±0.36)	0.57 (±0.35)	0.745	0.48	0.4
			STSG	Normal	54	0.61 (±0.36)	1.02 (±0.42)	<0.0001^*^	0.48	0.47
			Glyaderm®	Normal	54	0.57 (±0.35)	1.02 (±0.42)	<0.0001^*^	0.4	0.47
		6	STSG	Glyaderm®	56	0.64 (±0.34)	0.59 (±0.33)	0.963	0.49	0.47
			STSG	Normal	56	0.64 (±0.34)	1.02 (±0.38)	<0.0001^*^	0.49	0.47
			Glyaderm®	Normal	56	0.59 (±0.33)	1.02 (±0.38)	<0.0001^*^	0.47	0.47
		9	STSG	Glyaderm®	52	0.59 (±0.31)	0.58 (±0.30)	>0.9999	0.39	0.52
			STSG	Normal	52	0.59 (±0.31)	0.97 (±0.33)	<0.0001^*^	0.39	0.47
			Glyaderm®	Normal	52	0.58 (±0.30)	0.97 (±0.33)	<0.0001^*^	0.52	0.47
		12	STSG	Glyaderm®	58	0.63 (±0.35)	0.67 (±0.33)	0.1900	0.44	0.48
			STSG	Normal	58	0.63 (±0.35)	0.97 (±0.38)	<0.0001^*^	0.44	0.44
			Glyaderm®	Normal	58	0.67 (±0.33)	0.97 (±0.38)	0.0004^*^	0.48	0.44
	*R*2	3	STSG	Glyaderm®	54	0.83 (±0.07)	0.82 (±0.06)	>0.9999	0.11	0.1
			STSG	Normal	54	0.83 (±0.07)	0.74 (±0.13)	<0.0001^−^	0.11	0.15
			Glyaderm®	Normal	54	0.82 (±0.06)	0.74 (±0.13)	0.0008^+^	0.1	0.15
		6	STSG	Glyaderm®	56	0.81 (±0.09)	0.81 (±0.08)	NA	0.1	0.09
			STSG	Normal	56	0.81 (±0.09)	0.81 (±0.12)	NA	0.1	0.16
			Glyaderm®	Normal	56	0.81 (±0.08)	0.81 (±0.12)	NA	0.09	0.16
		9	STSG	Glyaderm®	52	0.81 (±0.08)	0.81 (±0.08)	NA	0.1	0.11
			STSG	Normal	52	0.81 (±0.08)	0.80 (±0.13)	NA	0.1	0.17
			Glyaderm®	Normal	52	0.81 (±0.08)	0.80 (±0.13)	NA	0.11	0.17
		12	STSG	Glyaderm®	58	0.81 (±0.09)	0.81 (±0.09)	NA	0.11	0.09
			STSG	Normal	58	0.81 (±0.09)	0.80 (±0.13)	NA	0.11	0.15
			Glyaderm®	Normal	58	0.81 (±0.09)	0.80 (±0.13)	NA	0.09	0.15
	*R*8	3	STSG	Glyaderm®	54	0.51 (±0.29)	0.47 (±0.28)	>0.9999	0.39	0.36
			STSG	Normal	54	0.51 (±0.29)	0.79 (±0.35)	<0.0001^*^	0.39	0.49
			Glyaderm®	Normal	54	0.47 (±0.28)	0.79 (±0.35)	<0.0001^*^	0.36	0.49
			6	STSG	Glyaderm®	56	0.50 (±0.26)	0.47 (±0.25)	>0.9999	0.37	0.34
	STSG			Normal	56	0.50 (±0.26)	0.82 (±0.33)	<0.0001^*^	0.37	0.48
	Glyaderm®		Normal	56	0.47 (±0.25)	0.82 (±0.33)	<0.0001^*^	0.34	0.48
	9		STSG	Glyaderm®	52	0.47 (±0.23)	0.45 (±0.22)	0.661	0.31	0.37
			STSG	Normal	52	0.47 (±0.23)	0.77 (±0.31)	<0.0001^*^	0.31	0.39
			Glyaderm®	Normal	52	0.45 (±0.22)	0.77 (±0.31)	<0.0001^*^	0.37	0.39
	12		STSG	Glyaderm®	58	0.51 (±0.25)	0.55 (±0.28)	>0.9999	0.38	0.4
			STSG	Normal	58	0.51 (±0.25)	0.77 (±0.34)	<0.0001^*^	0.38	0.42
			Glyaderm®	Normal	58	0.55 (±0.28)	0.77 (±0.34)	<0.0001^*^	0.4	0.42
Corneometer	Hydration	3	STSG	Glyaderm®	54	29.00 (±11.90)	27.72 (±12.37)	NA	15.97	18.63
			STSG	Normal	54	29.00 (±11.90)	30.66 (±13.69)	NA	15.97	21.76
			Glyaderm®	Normal	54	27.72 (±12.37)	30.66 (±13.69)	NA	18.63	21.76
		6	STSG	Glyaderm®	57	31.24 (±12.65)	34.92 (±29.79)	NA	14.48	18.81
			STSG	Normal	57	31.24 (±12.65)	28.80 (±12.96)	NA	14.48	18.16
			Glyaderm®	Normal	57	34.92 (±29.79)	28.80 (±12.96)	NA	18.81	18.16
		9	STSG	Glyaderm®	51	32.41 (±12.47)	32.52 (±12.98)	NA	13.08	16.26
			STSG	Normal	51	32.41 (±12.47)	29.20 (±12.36)	NA	13.08	19.34
			Glyaderm®	Normal	51	32.52 (±12.98)	29.20 (±12.36)	NA	16.26	19.34
		12	STSG	Glyaderm®	61	30.40 (±12.43)	30.91 (±13.75)	NA	16.48	15.34
			STSG	Normal	61	30.40 (±12.43)	30.63 (±12.72)	NA	16.48	20.66
			Glyaderm®	Normal	61	30.91 (±13.75)	30.63 (±12.72)	NA	15.34	20.66
Tewameter	TEWL	3	STSG	Glyaderm®	50	12.12 (±3.62)	13.97 (±4.90)	NA	4.34	6.25
			STSG	Normal	50	12.12 (±3.62)	14.05 (±7.62)	NA	4.34	3.13
			Glyaderm®	Normal	50	13.97 (±4.90)	14.05 (±7.62)	NA	6.25	3.13
		6	STSG	Glyaderm®	56	12.29 (±6.14)	13.50 (±10.53)	NA	4.07	3.9
			STSG	Normal	56	12.29 (±6.14)	12.85 (±5.76)	NA	4.07	4.4
			Glyaderm®	Normal	56	13.50 (±10.53)	12.85 (±5.76)	NA	3.9	4.4
		9	STSG	Glyaderm®	51	12.93 (±7.83)	12.79 (±5.85)	NA	5.88	4.15
			STSG	Normal	51	12.93 (±7.83)	12.01 (±3.79)	NA	5.88	3.95
			Glyaderm®	Normal	51	12.79 (±5.85)	12.01 (±3.79)	NA	4.15	3.95
		12	STSG	Glyaderm®	61	13.32 (±9.14)	13.01 (±6.53)	NA	5.55	5.25
			STSG	Normal	61	13.32 (±9.14)	13.70 (±6.35)	NA	5.55	6.3
			Glyaderm®	Normal	61	13.01 (±6.53)	13.70 (±6.35)	NA	5.25	6.3

*NA* Not applicable due to no significant differences present among groups as shown with multiple comparisons testing in Supplementary S5 (see online supplementary materials). Significant results are indicated in bold: + significance in favour of the intervention site; − significance in favour of the control site; ^*^ significance in favour of normal skin. *n* number of patients, *IQR* interquartile range (IQR) = Q3–Q1, *SD* standard deviation. Comparisons concerning the Mexameter control *vs.* intervention group: significant pigmentation/erythema in favour of the control group or intervention group is having an erythema/pigmentation index more in line with the values of normal skin than the other group. ^a^Indicates a Tukey’s test for statistical analysis, otherwise a Dunn’s test was used; Corneometer and Mexameter result are not included in this table given that there were no significant differences among groups

##### Tewameter TM 300

Mean (±SD) TEWL values of SoC, Glyaderm® and normal skin at 12 months follow-up were 13.32 (±9.18), 13.01 (±6.53) and 13.70 (±6.35) g/h/m^2^, respectively. There were no differences between groups (SoC, Glyaderm® and normal skin) at 3, 6, 9 and 12 months follow-up.

##### Mexameter MX 18

Mean (±SD) EI values of SoC, Glyaderm® and normal skin at 12 months follow-up were 359.14 (±98.64), 368.68 (±86.70) and 291.38 (±84.20) EI, respectively. There were no differences between the two treatment groups (SoC and Glyaderm®) at 3, 6, 9 and 12 months follow-up. At every follow-up, there was a significant difference in EI between treated areas and normal skin.

Mean (±SD) MI values of SoC, Glyaderm® and normal skin at 12 months follow-up were 221.75 (±149.69), 215.68 (±127.37) and 238.50 (±115.18) MI, respectively. At 12 months follow-up there was no significant difference in the control group compared to the intervention group nor was there a difference (borderline) in the control and intervention group compared to the pigmentation values of normal skin (*p* = 0.056).

##### Cutometer MPA 580

Mean (±SD) *R*0 values of SoC, Glyaderm® and normal skin at 12 months follow-up were 0.63 (±0.35), 0.67 (±0.33) and 0.97 (±0.38) respectively. There were no differences between the control and intervention groups at any follow-up. At every follow-up, both the control and intervention groups had significantly lower *R*0 values compared to those of normal skin (*p* < 0.05).

Mean (±SD) R2 values of SoC, Glyaderm® and normal skin at 12 months follow-up were 0.81 (±0.09), 0.81 (±0.09) and 0.80 (±0.13), respectively. At 3 months follow-up there were no significant differences in *R*2 values between the control and intervention groups. The *R*2 values of normal skin were significantly better than those of the control (*p* > 0.0001) or intervention group (*p* > 0.0001). There were no differences in *R*2 values between groups (SoC, Glyaderm® and normal skin) at 6, 9 and 12 months (*p* > 0.05) follow-up.

Mean (±SD) *R*8 values of SoC, Glyaderm® and normal skin at 12 months follow-up were 0.51 (±0.25), 0.55 (±0.28) and 0.77 (±0.34), respectively. There were no differences between the control and intervention groups at any follow-up. At every follow-up, both the control and intervention groups had significantly worse *R*0 values compared to those of normal skin (*p* < 0.05).

#### Subjective measurements

The number of patients, mean values and corresponding SDs, statistical tests and complementary statistics of all the subjective measurements can be found in Table 4.

**Table 4:** Pairwise comparisons subjective measurements

**Subjective parameters**																	
		**3 months**				**6 months**				**9 months**				**12 months**			
		***n***	**STSG**	**Glyaderm**	***P*** **-value**	***n***	**STSG**	**Glyaderm**	***P***-**value**	***n***	**STSG**	**Glyaderm**	***P*** **-value**	***n***	**STSG**	**Glyaderm**	***P*** **-value**
Observer	Vascularity	62	3.79 (±1.46)	4.03 (±1.41)	0.122	60	3.38 (±1.43)	3.63 (±1.40)	0.059	54	3.28 (±1.39)	3.22 (±1.36)	0.643	66	2.86 (±1.19)	3.09 (±1.31)	0.072
	Pigmentation	62	3.66 (±1.21)	3.79 (±1.38)	0.298	59	3.31 (±1.37)	3.55 (±1.35)	0.042^−^	54	2.88 (±0.88)	2.94 (±0.94)	0.699	66	2.91 (±1.08)	3.21 (±1.17)	0.010^−^
	Thickness	62	3.55 (±1.11)	3.55 (±1.36)	0.926	60	3.32 (±1.56)	3.43 (±1.29)	0.393	54	3.00 (±1.20)	2.80 (±1.09)	0.094	66	2.86 (±1.28)	2.91 (±1.20)	0.785
	Relief	62	3.52 (±1.21)	3.58 (±1.36)	0.725	60	3.22 (±1.50)	3.38 (±1.43)	0.297	54	3.06 (±1.25)	2.85 (±1.07)	0.183	66	2.86 (±1.29)	2.98 (±1.12)	0.330
	Pliability	61	4.03 (±1.60)	3.92 (±1.75)	0.740	59	3.29 (±1.46)	3.53 (±1.38)	0.065	54	3.02 (±1.45)	2.87 (±1.40)	0.352	66	2.74 (±1.22)	2.91 (±1.21)	0.131
	Surface	62	3.37 (±1.16)	3.35 (±1.29)	0.933	60	3.03 (±1.47)	3.05 (±1.27)	0.993	54	2.69 (±1.27)	2.69 (±1.31)	0.930	66	2.61 (±1.05)	2.71 (±1.03)	0.363
	Overall opinion	57	3.64 (±1.13)	3.68 (±1.28)	0.708	57	3.16 (±1.37)	3.37 (±1.17)	0.158	54	3.09 (±1.23)	2.98 (±1.21)	0.336	64	2.73 (±1.07)	3.02 (±1.05)	0.013^−^
Patient	Pain	60	2.52 (±2.25)	2.42 (±1.89)	0.897	62	2.27 (±1.89)	2.10 (±1.53)	0.345	55	2.00 (±1.68)	1.96 (±1.53)	0.881	65	1.88 (±1.60)	1.77 (±1.26)	0.446
	Itch	60	3.42 (±2.57)	3.67 (±2.65)	0.321	61	3.33 (±2.46)	3.43 (±2.55)	0.912	54	2.81 (±2.02)	2.76 (±1.95)	0.735	65	2.72 (±2.98)	2.52 (±2.05)	0.694
	Colour	59	6.78 (±1.81)	6.93 (±1.96)	0.313	61	5.85 (±2.17)	6.69 (±9.37)	0.158	54	5.44 (±1.91)	5.19 (±1.79)	0.112	65	4.68 (±1.95)	4.55 (±2.02)	0.174
	Rigidity	60	6.53 (±1.84)	6.80 (±1.97)	0.224	61	5.89 (±2.07)	5.74 (±2.11)	0.488^a^	55	4.87 (±2.05)	5.00 (±2.15)	0.546^a^	65	4.91 (±2.16)	5.15 (±2.27)	0.301
	Thickness	60	6.22 (±1.89)	6.43 (±2.02)	0.295	61	5.46 (±2.33)	5.18 (±2.26)	0.318	55	4.76 (±2.29)	4.64 (±2.27)	0.436	65	4.82 (±2.34)	4.74 (±2.12)	0.534
	Bumpiness	60	5.60 (±2.29)	5.77 (±2.28)	0.658	62	5.06 (±2.47)	4.74 (±2.35)	0.331	55	4.69 (±2.27)	4.31 (±2.00)	0.118	65	4.57 (±2.38)	4.58 (±2.07)	0.967
	General impression	60	5.13 (±2.24)	5.35 (±2.28)	0.393	62	4.65 (±1.84)	4.45 (±1.71)	0.223	55	4.53 (±2.00)	4.07 (±1.80)	0.005^+^	65	4.31 (±1.91)	4.03 (±1.85)	0.029^+^
AVSS	Colour	62	1.60 (±0.86)	1.63 (±0.81)	0.801	62	1.31 (±0.92)	1.39 (±0.95)	0.471	54	1.22 (±0.98)	1.19 (±0.93)	0.829	66	1.06 (±0.97)	0.98 (±0.94)	0.610
	Pliability	62	1.69 (±0.74)	1.65 (±0.68)	0.699	62	1.44 (±0.90)	1.40 (±0.82)	0.861	54	1.15 (±0.90)	1.17 (±0.93)	0.951	66	1.06 (±0.89)	1.18 (±0.84)	0.216
	Height	62	0.94 (±0.44)	0.98 (±0.53)	0.590	62	0.82 (±0.22)	0.85 (±0.60)	0.774	54	0.78 (±0.66)	0.72 (±0.60)	0.494	66	0.73 (±0.57)	0.80 (±0.51)	0.269
	Defects	62	0.07 (±0.25)	0.02 (±0.13)	0.375	62	0.05 (±0.22)	0.06 (±0.25)	>0.999	54	0.00 (±0.00)	0.00 (±0.00)	(^*^)	64	0.03 (±0.18)	0.02 (±0.13)	>0.999
	Itch	62	0.63 (±0.83)	0.65 (±0.87)	>0.999	62	0.65 (±0.85)	0.79 (±0.94)	0.056	54	0.43 (±0.60)	0.41 (±0.60)	>0.999	66	0.53 (±0.81)	0.42 (±0.66)	0.125
	Pigmentation	62	1.98 (±0.59)	2.02 (±0.61)	0.745	62	1.58 (±0.90)	1.73 (±0.81)	0.202	54	1.65 (±0.78)	1.71 (±0.77)	0.640	66	1.77 (±0.72)	1.80 (±0.71)	0.782
	Total	62	6.92 (±1.74)	6.95 (±1.85)	0.606	62	5.87 (±2.63)	6.24 (±2.58)	0.123^a^	54	5.22 (±2.43)	5.17 (±2.46)	0.844^a^	66	5.11 (±2.47)	5.09 (±2.40)	0.957^a^

^a^A paired t-test was used, otherwise a paired Wilcoxon test was used for statistical testing. (^*^) Pairwise comparison not possible due to mean difference between groups is equal to zero. Significant results are indicated in bold: + significance in favour of intervention site, – significance in favour of the control site. Number of patients (n), mean values and standard deviations of the subjective measurements

##### AVSS

No significant differences were found in any of the individual parameters nor the total score of the AVSS at 3, 6, 9 or 12 months follow-up between the control group and the intervention group (*p* > 0.05) (Table 4).

##### POSAS

###### POSAS observer

At 12 months follow-up, all the parameters were comparable in both groups except for pigmentation and the overall score. There was a significant worse score in terms of pigmentation and overall opinion for the intervention group (*p* = 0.010 and *p* = 0.013) (Table 4).

###### POSAS patient

**Figure 13:**
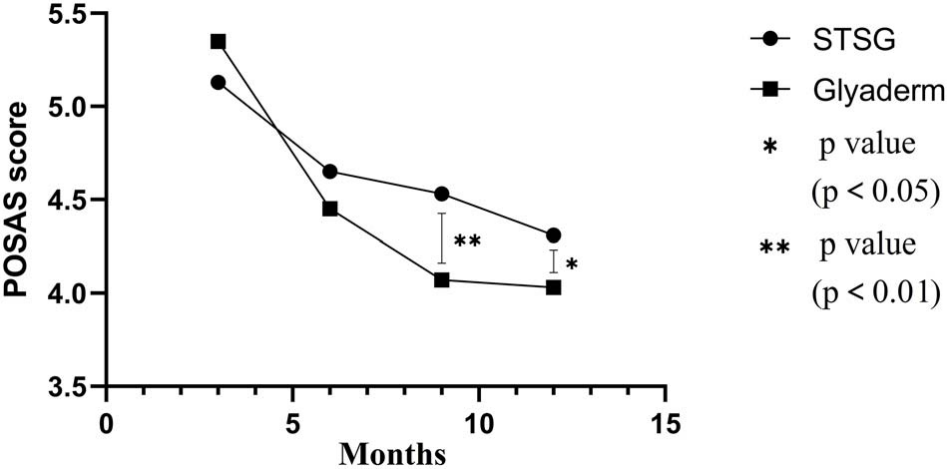
POSAS patient. Overall opinion as subjectively attributed by blinded patients using the POSAS at different time intervals during the follow-up period of 1 year after wound closure for both treatments; ^*^ significant difference, ^*^^*^ strong significant difference. *POSAS* Patient and Observer Scar Assessment Scale

At both 9 and 12 months follow-up there was a significant difference in terms of overall opinion, in favour of the group that was treated with Glyaderm® (*p* = 0.005 and *p* = 0.013, respectively) (Figure 13). The other individual parameters were comparable between the control and intervention groups and were comparable at every follow-up (Table 4).

#### Biopsies

**Figure 14:**
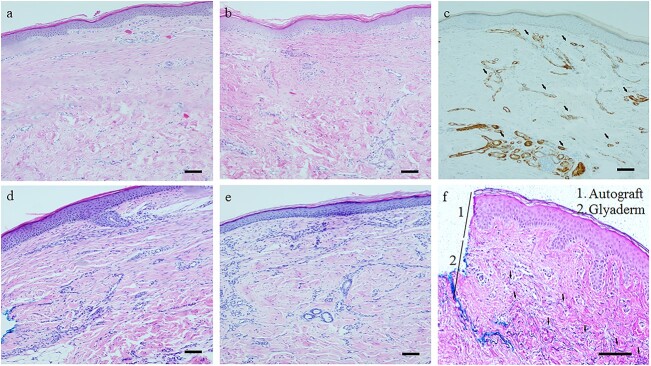
Light microscopy of histological slices. (**a**) Hematoxylin and eosin (H&E) histological slice of control site at 3 months follow-up. (**b**) H&E histological slice of intervention site at 3 months follow-up. (**c**) Alpha-smooth muscle actin staining of histological slice of the intervention group at 3 months follow-up. Arrows indicate vascular structures in the papillar and reticular dermis indicating a well-vascularized neo-dermis. (**d**) H&E histological slice of control site at 12 months follow-up. (**e**) H&E histological slice of intervention site at 12 months follow-up. (**f**) Elastica Von Giesson staining visualizing the presence of donor elastin fibres at 12 months follow-up; autograft and Glyaderm® are indicated. Single arrows indicate some example donor elastin fibres in the deep dermis. The elastic fibres are coloured blue due to the staining procedure. Scale bars of 100 μm are indicated in the right lower corner of each individual image

The number of patients, mean values, corresponding SDs and complementary statistics of the histological scores can be found in Supplementary S6, see online supplementary material. No statistically significant differences could be found for the biopsies of the control group *vs.* the intervention group at 3 and 12 months (Figure 14). However, 57 out of 58 of the sites treated with Glyaderm® clearly showed the presence of donor elastin fibres at 12 months after wound healing (Figure 14f), illustrating the longevity of the fibres. The presence of elastin was characterized by a histological score ≤4. A satisfying number of elastin fibres of favourable quality, characterized by a histological score ≤3, were seen in 34 out of 58 biopsies.

## Discussion

Early debridement and immediate coverage of extensive skin defects with STSG is essential for the survival of patients with severe burns, but the lack of dermis often results in HTS formation and contractures [18]. In the case of extensive full-thickness burn wounds, the dermal layer cannot be reconstructed using the classical reconstructive approaches, e.g. full-thickness skin grafts or flaps. A possible alternative is to use dermal substitutes with immediate or delayed autografting [36]. In this study, we investigated the short- and long-term cosmetic and functional outcomes following the use of Glyaderm® in a single-stage procedure.

The major advantage of acellular dermal templates derived from human allograft skin such as Glyaderm® is that they retain the native dermal structure, with the characteristics of the natural porosities required for dermal regeneration, vascularization and innervation [20,26,37]. When combining a dermal substitute and autograft in a single operation, the main limiting factor is inadequate vascularization, risking partial or complete necrosis of both substitute and autograft [38]. Most DRTs are applied in a two-step procedure, with autografting being delayed for several weeks to ensure incorporation and vascularization of the inherently avascular substitute [7]. However, the literature confirms that dermal replacement and coverage with skin grafts, primarily due to shrinking health care resources, should ideally be performed in a single-stage procedure if autografts are available [39]. In general the thickness of DRTs ranges from 0.040–0.080 inches (1–2 mm) [36]. The restricted and standardized thickness of Glyaderm® (0.012 inches or 0.30 mm) grants fast and adequate neovascularization and allows a one-stage procedure, illustrated by the excellent average graft take of 96.22%, combined with only a limited SD (± 5.40), achieved in this study [36].

Research has shown that alterations in both elastin organization and content contribute to the formation of scars [25]. A key component of Glyaderm® is the natural collagen–elastin matrix in which elastin fibres with microfibrils are incorporated and well-preserved even after decellularization [27,28,40]. The incorporation of elastin either acts as a replacement or promotes the synthesis of elastin fibres, as is seen in animal models where the use of these collagen–elastin scaffolds can even induce a limited level of elastin fibre deposition, whereas collagen-only scaffolds do not [20,26,41,42]. The presence of elastin interrupts myofibroblast differentiation and therefore less collagen contraction is observed, leading to improved elasticity of the scar [1]. By means of skin biopsies, this study demonstrates the longevity of the donor elastin fibres, and improvement of scar elasticity was shown with improved overall opinion on the patient POSAS. The collagen fibres present in xenografts or synthetic grafts have often been chemically cross-linked to enhance stability and decrease susceptibility to early degradation [18,43]. Expedited degradation is unfavourable due to the potential risk of increased fibrosis [44]. However, early degradation is not desirable, but having no implant degradation can impede cellular activity *in situ* [44]. Due to the cytotoxicity of the cross-linking chemicals, adverse effects on host response might be considerable [18,43]. In contrast, non-cross-linked templates, such as Glyaderm®, are well tolerated and stimulate tissue regeneration in addition to minimal inflammatory responses, whilst still respecting one of the main principles of reconstructive surgery: replacing ‘like with like’ [18,21,43].

The price of the two most well-known collagen-elastin acellular dermal matrices (ADMs), the human-derived Alloderm® and bovine-derived Matriderm, is respectively €30/cm^2^ ([45] price 2012) and €5.02/cm^2^ (0.080 inches/2 mm) (price 2022) [46–48]. However, Alloderm’s use is limited in the reconstruction of burn injuries. Glyaderm® costs €4.74/cm^2^ (price 2022) and has lower costs compared to other biological collagen–elastin DRTs [46]. Prices in this article were obtained through representatives, with the exception of the price of Alloderm which was obtained through the literature. All given prices are target prices and depend on, e.g. order quantity, substitute dimensions and the hospital. However, the best known and probably most widely used DRT is Integra, an ~0.030 inches/0.80 mm (€16.36/cm^2^ price 2022) thick bilaminar cross-linked bovine-derived collagen-based dermal matrix requiring a two-stage procedure [1,4,49]. The necessity of a two-stage procedure, high risk of infection, inconsistent long-term results, absence of elastin and the huge financial burden are the most reported drawbacks and are therefore important limiting factors for general use [4,49]. The problem with synthetic bilayers is the difficult initial wound adherence and fluid accumulation which leads to the development of seromas and harbours an increased risk of infection, which is the most frequent complication seen with Integra [37,49]. A recent paper published by Gonzalez *et al*. reviewed 26 studies reporting infection rates with the use of Integra [49]. The research group stated that on average 16.9% of Integra-engrafted sites led to infection [49]. In this study, none of the Glyaderm® nor the covering autografts were lost due to (major) infection. Although the two-stage technique is deemed to be reliable, it also necessitates a treatment period that is prolonged to several weeks to allow sufficient ingrowth of supporting blood vessels and requires additional operations and anaesthetic administrations [7]. Additionally a two-stage technique is subsequently associated with increased hospitalization time and a higher number of outpatient visits [7]. However, when confronted with limited availability of donor sites, temporary coverage by dermal substitutes in a two-stage procedure can be beneficial.

The substantial reductions in hospital length of stay, fewer operative encounters, and reduced outpatients visits and health-care expenditure, combined with the fact that infections might lead to potential loss of both the dermal substitute and covering graft, a significant decrease in costs can be expected when using DRTs capable being engrafted in a single-stage [7]. However, not all dermal substitutes allow for a successful one-stage procedure and require multiple surgeries for reconstruction. Integra Single layer ‘Thin’, is a 0.016 inches/0.40 mm (€12.2/cm^2^ price 2022) thick DRT that can be used in a one-stage setting, but lacks elastin fibres. Furthermore, bovine-derived Matriderm 1 mm single layer (€5.32/cm^2^ (price 2022)) and human-derived SureDerm (price not available) are two commercially available collagen–elastin DRTs that are being used in a one-stage setting.

In this single-blinded, randomized controlled trial, our research group demonstrated the successful applicability of simultaneous bilayered skin reconstruction using Glyaderm® as an acellular dermal substitute in patients with various full-thickness defects. There was no partial nor complete loss of the dermal substitute (Glyaderm)® due to inadequate vascularization nor infection, associated with nearly perfect graft take, comparable wound closure times. Although the thickness of Glyaderm® has been standardized and restricted to a thin 0.012 inch (0.30 mm) sheet, which is much thinner than most dermal substitutes, this acellular DRT led to increased overall scar quality based on the POSAS (overall opinion) of the patient and therefore most likely leads to improved patient satisfaction with his/her scar [36]. The majority of patients indicated that these more favourable results are due to a more normalized skin sensation at the site that received dermal replacement. Decreased donor-site morbidity and preserved sensory functioning has been reported with the use of dermal substitutes covering phalloplasty donor sites [50]. Watfa *et al*. investigated the effects of single-stage reconstruction with Matriderm® after radial forearm flap harvest and found that the group that was treated with the bilayered skin reconstruction had more preserved sensory nerve functioning and skin sensibility [50].

Compared to the two-stage procedure, a one-stage reconstruction with Glyaderm® did not deliver a statistically significant improvement in terms of scar elasticity [26]. However, the reduced thickness of the Glyaderm® sheet may provide less benefit in elasticity compared to a more substantial layer [39].

The ideal skin substitute should be inexpensive, effective, widely available, easy to produce, easy to transport and store, be of human origin and should have a low infection susceptibility, lack antigenicity, quickly adhere to the wound bed, protect the wound from dehydration, allow excellent graft take, activate and modulate the cicatrization process, should not be biodegradable too quickly and should finally but most importantly result in improved scar quality [19,51]. The results of previous extensive research combined with the outcomes of this high-level evidence study are favourable towards presuming that Glyaderm® is an ADM that meets most of these rigorous requirements [26–28,46,52,53].

### Limitations of the study

Part of the study period was during the COVID-19 pandemic and thus some patients could not receive their proper follow-up, leading to loss of valuable data. To counter this loss, the research group decided to include additional patients, raising the number of wound comparisons from the included 75, which was derived from power analysis, to 82.

## Conclusions

Combined with adequate debridement and proper wound bed preparation, a standardized thickness of 0.012-inch (0.30 mm) Glyaderm® enables the use of a single-stage procedure for deep and full-thickness skin defects, which is universally favoured by all surgeons. In contrast to most dermal substitutes available, no infections were seen and optimal graft take was achieved. Glyaderm® can thus be used as an ADM in the reconstruction of full-thickness burns or other comparable full-thickness defects, eventually resulting in long-term increased scar quality and therefore most likely patient satisfaction and improved quality of life.

## Abbreviations

ADM: Acellular dermal matrix; ANOVA: Analysis of variance; AVSS: Adapted Vancouver Scar Scale; DRTs: Dermal regeneration templates; EI: Erythema index; GPAs: Glycerol preserved allografts; HTS: Hypertrophic scar; LDI: Laser Doppler imaging; MI: melanin index; POSAS: Patient and Observer Scar Assessment Scale; RFF: Radial forearm flap; SD: Standard deviation; SoC: Standard of care; STSG: Split-thickness skin graft; TEWL: Transepidermal water loss.

## Funding

Research Foundation Flanders (FWO) supplied a grant within Applied Biomedical Research (TBM) for project number T001316N entitled: ‘*The application of Glyaderm® as a dermal substitute in the reconstruction of deep burns or other full-thickness skin defects*’.

## Data availability

All data are presented in the main manuscript. The CONSORT checklist can be found in online Supplementary S7. Supplementary data on the histological parameters can be found in online Supplementary S8.

## Authors’ contributions

All authors have made a substantial contribution to: the conception and design of the study, acquisition of data, analysis and interpretation of data, drafting the article, revising the article critically for important intellectual content and final approval of the version to be submitted.

## Ethics approval and consent to participate

The Ethical Review Committee of Ghent University Hospital approved this prospective study (Belgian registration number B670201733327). The trial was registered on clinicaltrials.gov and received the following registration code: NCT01033604.

## Consent for publication

All included patients and authors of this study gave consent for publication.

## Competing interests

None declared.

## Supplementary Material

Supplementary_material_1_tkad015

Supplementary_material_2_tkad015

Supplementary_material_3_tkad015

Supplementary_material_4_tkad015

Supplementary_material_5_tkad015

Supplementary_material_6_tkad015

Supplementary_material_7_tkad015

Supplementary_material_8_tkad015
