# Supplementary material for: A single-stage bilayered skin reconstruction using Glyaderm® as an acellular dermal regeneration template results in improved scar quality: an intra-individual randomized controlled trial
Source: Burns Trauma. 2023 May 2;11:tkad015. doi: 10.1093/burnst/tkad015 (PMC10152996; doi:10.1093/burnst/tkad015)
Supplement: Supplementary_material_1_tkad015 [file supplementary_material_1_tkad015.pdf]

# POSAS Observer scale

The Patient and Observer Scar Assessment Scale v2.0 / EN

**Date of examination:**

**Observer:**

**Location:**

**Research / study:**

**Name of patient:**

**Date of birth:**

**Identification number:**

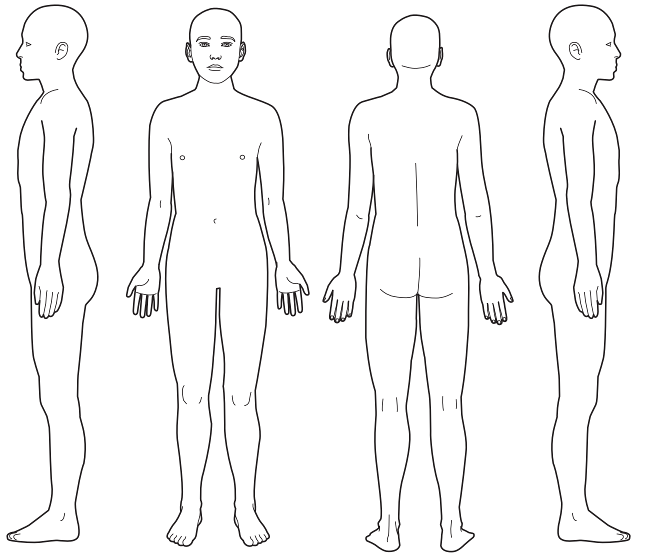

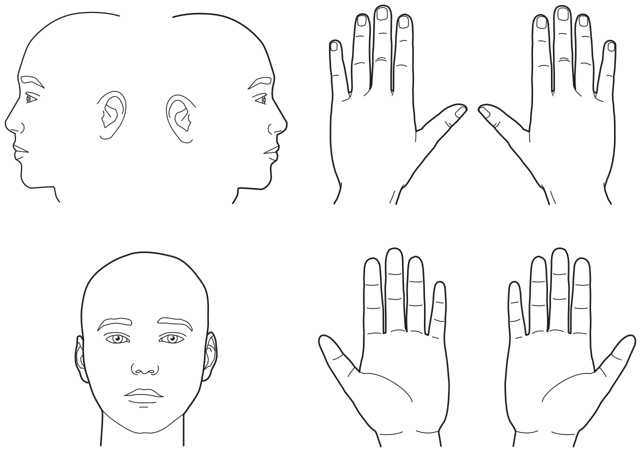

**1 = normal skin** **worst scar imaginable = 10**

| PARAMETER | 1 | 2 | 3 | 4 | 5 | 6 | 7 | 8 | 9 | 10 | CATEGORY |
|---|---|---|---|---|---|---|---|---|---|---|---|
| VASCULARITY | | | | | | | | | | | PALE \| PINK \| RED \| PURPLE \| MIX |
| PIGMENTATION | | | | | | | | | | | HYPO \| HYPER \| MIX |
| THICKNESS | | | | | | | | | | | THICKER \| THINNER |
| RELIEF | | | | | | | | | | | MORE \| LESS \| MIX |
| PLIABILITY | | | | | | | | | | | SUPPLE \| STIFF \| MIX |
| SURFACE AREA | | | | | | | | | | | EXPANSION \| CONTRACTION \| MIX |
| OVERALL OPINION | | | | | | | | | | | |

## Explanation

The observer scale of the POSAS consists of six items (vascularity, pigmentation, thickness, relief, pliability and surface area).
All items are scored on a scale ranging from 1 ('like normal skin') to 10 ('worst scar imaginable').
The sum of the six items results in a total score of the POSAS observer scale. Categories boxes are added for each item. Furthermore, an overall opinion is scored on a scale ranging from 1 to 10.
All parameters should preferably be compared to normal skin on a comparable anatomic location.

## Explanatory notes on the items:

- **VASCULARITY** Presence of vessels in scar tissue assessed by the amount of redness, tested by the amount of blood return after blanching with a piece of Plexiglas
- **PIGMENTATION** Brownish coloration of the scar by pigment (melanin); apply Plexiglas to the skin with moderate pressure to eliminate the effect of vascularity
- **THICKNESS** Average distance between the subcutical-dermal border and the epidermal surface of the scar
- **RELIEF** The extent to which surface irregularities are present (preferably compared with adjacent normal skin)
- **PLIABILITY** Suppleness of the scar tested by wrinkling the scar between the thumb and index finger
- **SURFACE AREA** Surface area of the scar in relation to the original wound area

COPYRIGHT © P.P.M. VAN ZUIJLEN, BEVERWIJK-NL

# POSAS Patient scale

The Patient and Observer Scar Assessment Scale  v2.0 / EN

**Date of examination:**

**Observer:**

**Location:**

**Research / study:**

**Name of patient:**

**Date of birth:**

**Identification number:**

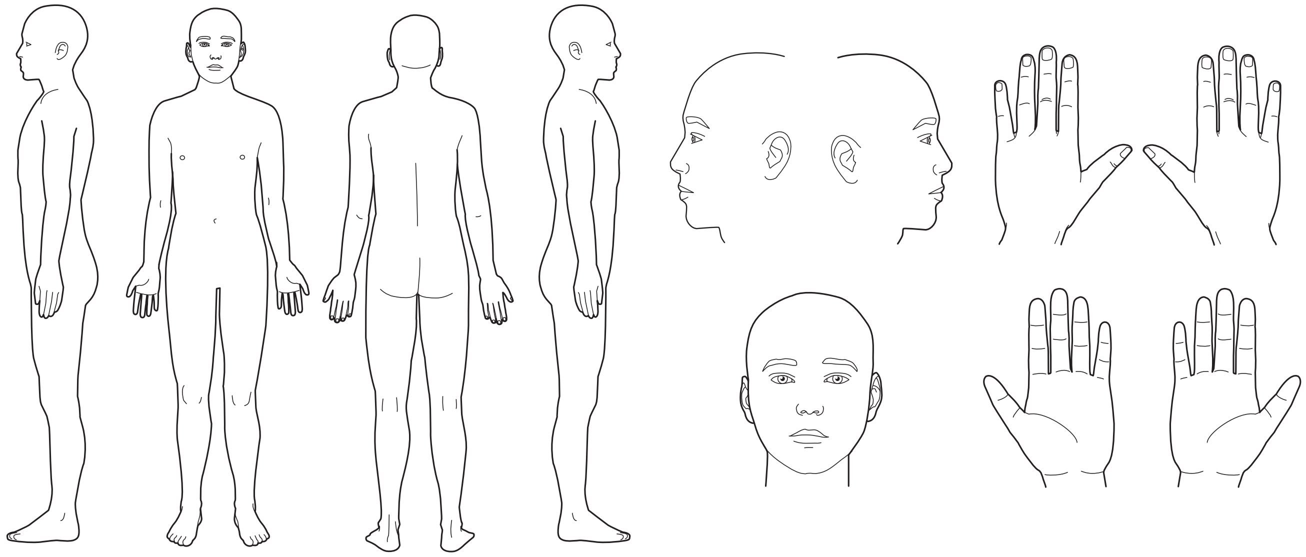

| | 1 = no, not at all | | | | | | | | yes, very much = 10 |
|---|---|---|---|---|---|---|---|---|---|---|
| | 1 | 2 | 3 | 4 | 5 | 6 | 7 | 8 | 9 | 10 |
| HAS THE SCAR BEEN PAINFUL THE PAST FEW WEEKS? | ○ | ○ | ○ | ○ | ○ | ○ | ○ | ○ | ○ | ○ |
| HAS THE SCAR BEEN ITCHING THE PAST FEW WEEKS? | ○ | ○ | ○ | ○ | ○ | ○ | ○ | ○ | ○ | ○ |

| | 1 = no, as normal skin | | | | | | | | yes, very different = 10 |
|---|---|---|---|---|---|---|---|---|---|---|
| | 1 | 2 | 3 | 4 | 5 | 6 | 7 | 8 | 9 | 10 |
| IS THE SCAR COLOR DIFFERENT FROM THE COLOR OF YOUR NORMAL SKIN AT PRESENT? | ○ | ○ | ○ | ○ | ○ | ○ | ○ | ○ | ○ | ○ |
| IS THE STIFFNESS OF THE SCAR DIFFERENT FROM YOUR NORMAL SKIN AT PRESENT? | ○ | ○ | ○ | ○ | ○ | ○ | ○ | ○ | ○ | ○ |
| IS THE THICKNESS OF THE SCAR DIFFERENT FROM YOUR NORMAL SKIN AT PRESENT? | ○ | ○ | ○ | ○ | ○ | ○ | ○ | ○ | ○ | ○ |
| IS THE SCAR MORE IRREGULAR THAN YOUR NORMAL SKIN AT PRESENT? | ○ | ○ | ○ | ○ | ○ | ○ | ○ | ○ | ○ | ○ |

| | 1 = as normal skin | | | | | | | | very different = 10 |
|---|---|---|---|---|---|---|---|---|---|---|
| | 1 | 2 | 3 | 4 | 5 | 6 | 7 | 8 | 9 | 10 |
| WHAT IS YOUR OVERALL OPINION OF THE SCAR COMPARED TO NORMAL SKIN? | ○ | ○ | ○ | ○ | ○ | ○ | ○ | ○ | ○ | ○ |

COPYRIGHT © P.P.M. VAN ZUIJLEN, BEVERWIJK-NL
