# Supplementary material for: A single-stage bilayered skin reconstruction using Glyaderm® as an acellular dermal regeneration template results in improved scar quality: an intra-individual randomized controlled trial
Source: Burns Trauma. 2023 May 2;11:tkad015. doi: 10.1093/burnst/tkad015 (PMC10152996; doi:10.1093/burnst/tkad015)
Supplement: Supplementary_material_2_tkad015 [file supplementary_material_2_tkad015.pdf]

Adapted Vancouver Scar scale (AVSS)

Subdivision	Score	3 months	6 months	9 months	12 months
**A. Color**					
Normal skin colour	**0**				
Pink	**1**				
Red	**2**				
purple	**3**				
**B. Pliability**					
Normal	**0**				
Supple-min resist	**1**				
Yielding-moderate	**2**				
Firm-inflexible	**3**				
Banding-blanches	**4**				
**C. Height**					
Normal	**0**				
< 2mm	**1**				
2 – 5mm	**2**				
> 5mm	**3**				
**D. Defects**					
Absent	**0**				
Present	**1**				
**E. Itch**					
No itching	**0**				
Slight itching	**1**				
Moderate itching	**2**				
Serious itching	**3**				
**F. Pigmentation**					
Normal	**0**				
Hypo-pigmentation	**1**				
Mixed	**2**				
Hyper-pigmentation	**3**				
**TOTAL SCORE**					
