# Supplementary material for: A single-stage bilayered skin reconstruction using Glyaderm® as an acellular dermal regeneration template results in improved scar quality: an intra-individual randomized controlled trial
Source: Burns Trauma. 2023 May 2;11:tkad015. doi: 10.1093/burnst/tkad015 (PMC10152996; doi:10.1093/burnst/tkad015)
Supplement: Supplementary_material_3_tkad015 [file supplementary_material_3_tkad015.docx]

| HISTOLOGICAL PARAMETER | HISTOLOGICAL SCORE | LEGEND |
| --- | --- | --- |
|  |  |  |
| Organisation of collagen fibers | 0 | Normal |
|  | 1 - 4 | Intermediate |
|  | 5 | Strong broadening and eosinophilic bundles (keloid-like) |
| The general view and aspect of the epidermis | 0 | Normal |
|  | 1 - 4 | Intermediate |
|  | 5 | Complete disappearing of rete ridges |
| Degree of inflammation / type of white blood cells | 0 | No inflammation |
|  | 1 - 4 | Intermediate |
|  | 5 | Strong dermal inflammation |
| Number of myofibroblasts | 1 | Absent |
|  | 1 - 4 | Intermediate |
|  | 5 | Entire dermis positive for SMA staining |
| Blood vessel organisation | 0 | Normal |
|  | 1 - 4 | Intermediate |
|  | 5 | Strong increase and verticalisation of blood vessels |
| Organisation and amount of elastin | 0 | Normal |
|  | 1 - 4 | Intermediate |
|  | 5 | Complete absence of elastin fibers |

*Supplementary material 3 – Histological scoring system*
