# Supplementary material for: A single-stage bilayered skin reconstruction using Glyaderm® as an acellular dermal regeneration template results in improved scar quality: an intra-individual randomized controlled trial
Source: Burns Trauma. 2023 May 2;11:tkad015. doi: 10.1093/burnst/tkad015 (PMC10152996; doi:10.1093/burnst/tkad015)
Supplement: Supplementary_material_4_tkad015 [file supplementary_material_4_tkad015.docx]

| Pain scores |  |  |  |  |  |
| --- | --- | --- | --- | --- | --- |
| Moment of assessment | **n** | **STSG  mean (± SD)** | **Glyaderm mean (± SD)** | **Score^a^** | **p-value^b,c^** |
| Post-operative day 2 | 45 | 2.47 (± 2.08) | 2.22 (± 1.82) | -61 | 0.147 |
| Post-operative day 4 | 49 | 2.19 (± 1.98) | 2.28 (± 1.82) | 11 | 0.862 |
| Post-operative day 5/6/7  (Surfasoft removal) | 53 | 2.78 (± 2.04) | 3.01 (± 2.33) | 27 | 0.403 |
| Post-operative week 2 | 36 | 1.92 (± 1.99) | 2.03 (± 1.98) | 26 | 0.460 |
| Post-operative week 3 | 28 | 1.21 (± 1.57) | 1.14 (± 1.43) | -3 | >0.999 |
| Post-operative week 4 | 24 | 1.00 (± 1.38) | 1.25 (± 1.75) | 6 | 0.250 |
| Post-operative week 5 | 16 | 0.69 (± 0.87) | 1.50 (± 1.83) | 21 | 0.031 |
| Post-operative week 6 | 10 | 1.80 (± 1.93) | 1.90 (± 1.85) | 2 | >0.999 |
| Post-operative week 7 | 5 | 0.60 (± 0.89) | 0.60 (± 0.89) | NA^d^ | NA^d^ |
| a Score used by the Wilcoxon test (sum of signed ranks).  b The non-parametric Wilcoxon test was used for statistical analysis.  c Statistical significant if p ≤ 0.05.  d Statistical analysis not possible due to no difference in means. All recordings (n) had the same score. | | | | | |

*Supplementary material 4 – Pain scores (n= number of assessed wound comparisons). SD= standard deviation.*
