# Supplementary material for: A single-stage bilayered skin reconstruction using Glyaderm® as an acellular dermal regeneration template results in improved scar quality: an intra-individual randomized controlled trial
Source: Burns Trauma. 2023 May 2;11:tkad015. doi: 10.1093/burnst/tkad015 (PMC10152996; doi:10.1093/burnst/tkad015)
Supplement: Supplementary_material_5_tkad015 [file supplementary_material_5_tkad015.docx]

| Multiple comparisons objective measurements | | | | | | | | | | | | | | | | | | | | |  |
| --- | --- | --- | --- | --- | --- | --- | --- | --- | --- | --- | --- | --- | --- | --- | --- | --- | --- | --- | --- | --- | --- |
| Time | Corneometer | | | Tewameter | | | Mexameter | | | | | | Cutometer | | | | | | | | |
|  |  |  |  |  |  |  | Erythema | | | Pigmentation | | | R0 | | | R2 | | | R8 | | |
|  | n | p-value^c^ | score^d^ | n | p-value^c^ | score^d^ | n | p-value^c^ | score^d^ | n | p-value^c^ | score^d^ | n | p-value^c^ | score^d^ | n | p-value^c^ | score^d^ | n | p-value^c^ | score^d^ |
| 3m | 54 | 0.800^a^ | 0.444 | 50 | 0.156^a^ | 3.72 | 54 | **<0.0001**^b^ | 86.73 | 54 | **<0.0001**^a^ | 23.81 | 54 | **<0.0001^a^** | 50.33 | 54 | **<0.0001^a^** | 23.62 | 54 | **<0.0001^a^** | 38.21 |
| 6m | 57 | 0.343^a^ | 2.14 | 56 | 0.741^a^ | 0.601 | 57 | **<0.0001**^a^ | 46.46 | 57 | **<0.0001**^a^ | 19.89 | 56 | **<0.0001^a^** | 50.49 | 56 | 0.547**^a^** | 1.206 | 56 | **<0.0001^a^** | 53.14 |
| 9m | 51 | 0.087^b^ | 2.634 | 51 | 0.943^a^ | 0.178 | 52 | **<0.0001**^a^ | 41.69 | 52 | **0.001**^a^ | 13.88 | 52 | **<0.0001^a^** | 46.64 | 52 | 0.541**^a^** | 1.228 | 52 | **<0.0001^a^** | 44.69 |
| 12m | 61 | 0.527^a^ | 1.279 | 61 | 0.328^a^ | 2.23 | 61 | **<0.0001**^a^ | 43.18 | 61 | **0.018**^a^ | 8 | 58 | **<0.0001^a^** | 33.93 | 58 | 0.759**^a^** | 0.56 | 58 | **<0.0001^a^** | 33.96 |
| a The Friedman test was used for statistical analysis.  b The ANOVA test was used for statistical analysis  c Statistic used by the Friedman / ANOVA test.  d Statistical significant if p ≤ 0.05. | | | | | | | |  |  |  |  |  |  |  |  |  |  |  |  |  |  |

*Supplementary material 5 – Multiple comparisons tests for objective measurements*
