# Supplementary material for: A single-stage bilayered skin reconstruction using Glyaderm® as an acellular dermal regeneration template results in improved scar quality: an intra-individual randomized controlled trial
Source: Burns Trauma. 2023 May 2;11:tkad015. doi: 10.1093/burnst/tkad015 (PMC10152996; doi:10.1093/burnst/tkad015)
Supplement: Supplementary_material_6_tkad015 [file supplementary_material_6_tkad015.docx]

| **Histological analysis** | | | | | |
| --- | --- | --- | --- | --- | --- |
|  | **n** | **STSG**  mean (± SD) | **Glyaderm®**  mean (± SD) | **Score^b^** | **p-value^a,c^** |
| Collagen organization | | | | | |
| *3 months follow-up*  *12 months follow-up*  Epidermal aspect | 58  58 | 2.76 (± 0.80)  2.72 (± 0.81) | 2.95 (± 0.76)  2.90 (± 0.85) | 165  133 | .061  .220 |
| *3 months follow-up*  *12 months follow-up*  Inflammation | 58  58 | 2.02 (± 1.88)  1.83 (± 1.93) | 1.81 (± 1.85)  1.60 (± 1.75) | -106  -73 | .410  .387 |
| *3 months follow-up*  *12 months follow-up*  Blood vessels organization | 58  58 | 1.60 (± 1.02)  1.14 (± 0.74) | 1.71 (± 0.90)  1.60 (± 1.75) | 118  117 | .428  .151 |
| *3 months follow-up*  *12 months follow-up*  Number of myofibroblasts | 58  58 | 2.52 (± 0.82)  2.45 (± 0.99) | 2.71 (± 0.82)  2.36 (± 0.81) | 160  -75 | .119  .534 |
| *3 months follow-up*  *12 months follow-up*  Organization of elastin fibers | 58  57 | 1.53 (± 1.52)  0.30 (± 0.94) | 1.71 (± 1.59)  0.28 (± 0.77) | 88  -1 | .359  .999 |
| *3 months follow-up*  *12 months follow-up* | 58  58 | 2.71 (± 1.24)  2.76 (± 1.14) | 2.60 (± 1.18)  2.76 (± 1.22) | -63  -5 | .527  .968 |
| a The non-parametric Wilcoxon test was used for statistical analysis.  b Score used by the Wilcoxon test (Sum of signed ranks).  c Statistical significant if p ≤ 0.05. | | | | | |

*Supplementary material 6 – Histological analysis of scar biopsies of the control and intervention sites. SD = standard deviation. n = number of analyzed wound comparisons.*
