# Supplementary material for: A single-stage bilayered skin reconstruction using Glyaderm® as an acellular dermal regeneration template results in improved scar quality: an intra-individual randomized controlled trial
Source: Burns Trauma. 2023 May 2;11:tkad015. doi: 10.1093/burnst/tkad015 (PMC10152996; doi:10.1093/burnst/tkad015)
Supplement: Supplementary_material_8_tkad015 [file supplementary_material_8_tkad015.docx]

| **Histological analysis** | | | | | |
| --- | --- | --- | --- | --- | --- |
|  | **n** | **STSG**  Q1 | **STSG**  Q3 | **Glyaderm** Q1 | **Glyaderm** Q3 |
| Collagen organization | | | | | |
| *3 months follow-up*  *12 months follow-up*  Epidermal aspect | 58  58 | 2.00 2.00 | 3.00 3.00 | 3.00 2.25 | 3.00 3.00 |
| *3 months follow-up*  *12 months follow-up*  Inflammation | 58  58 | 0.00 0.00 | 4.00 4.00 | 0.00 0.00 | 3.75 3.00 |
| *3 months follow-up*  *12 months follow-up*  Blood vessels organization | 58  58 | 1.00 1.00 | 2.00 1.00 | 1.00 1.00 | 2.00  2.00 |
| *3 months follow-up*  *12 months follow-up*  Number of myofibroblasts | 58  58 | 2.00 2.00 | 3.00 3.00 | 2.00 2.00 | 3.00 3.00 |
| *3 months follow-up*  *12 months follow-up*  Organization of elastin fibers | 58  57 | 0.00 0.00 | 3.00 0.00 | 0.00 0.00 | 3.00 0.00 |
| *3 months follow-up*  *12 months follow-up* | 58  58 | 2.00 2.00 | 4.00 4.00 | 2.00 2.00 | 4.00 4.00 |

*Supplementary material 8 – Number of patients (n), first quartile (Q1) and third quartile (Q3) of the histological data.*
